# Pneumothorax as adverse event in patients with lung metastases of soft tissue sarcoma treated with pazopanib: a single reference centre case series

**DOI:** 10.1186/2045-3329-4-14

**Published:** 2014-10-01

**Authors:** Arie J Verschoor, Hans Gelderblom

**Affiliations:** 1 Department of Clinical Oncology, Leiden University Medical Centre, Albinusdreef 2, Leiden, The Netherlands

**Keywords:** Soft tissue sarcoma, Pazopanib, Pneumothorax, Adverse event, Pleural metastases, Pulmonary metastases

## Abstract

**Background:**

Recently, the phase III PALETTE study introduced pazopanib (Votrient®) as treatment for adult patients with locally advanced or metastatic non-liposarcoma soft tissue sarcoma after prior treatment with doxorubicin and/or ifosfamide. Pneumothorax was reported as adverse event in 8 of 246 treated patients (3.3%) in that study. This case series presents the incidence and clinic of this complication in the Leiden University Medical Centre.

**Cases:**

Forty-three patients were treated with pazopanib of which six patients (14.0%) developed a pneumothorax. These six patients were treated for malignant peripheral nerve sheath tumour, angiosarcoma, synovial sarcoma, fibromyxomatoid sarcoma, pleomorphic sarcoma and endometrial stromal sarcoma. All six patients had subpleural pulmonary or pleural metastases at the start of pazopanib and the pneumothorax developed during or shortly after treatment with pazopanib and was difficult to treat.

**Discussion:**

The incidence reported by us is higher than the incidence in the PALETTE study. Trials with pazopanib in renal cell carcinoma, urothelial carcinoma and cervix carcinoma did not report pneumothorax as an adverse event, suggesting pneumothorax as a specific adverse event in soft tissue sarcoma patients treated with pazopanib. This may be related to the fact that there is often pleural metastatic involvement and cystic degeneration due to pazopanib treatment may add to the risk.

**Conclusion:**

The risk of an, often difficult to treat, pneumothorax during pazopanib therapy should be discussed with the patient before initiation of treatment for a pulmonary metastasized sarcoma and physicians should be alert to the occurrence of such an event.

## Background

Soft tissue sarcomas (STS) are rare mesenchymal tumours originating from visceral and connective tissue. This group of tumours accounts for approximately one percent of all malignancies and consists of more than 50 histological subtypes [1]. The only curative treatment is surgical resection with large margins with or without adjuvant radiotherapy [1]. Treatment for locally advanced and metastatic disease is usually palliative and was until recently mostly confined to anthracyclin or ifosfamide based chemotherapy, apart from specific chemotherapy regimens used for specific subgroups [1]. Reported response rates vary between 16-27% and median survival is reported to be 12 months [2]. Trabectedin was introduced recently for STS, mainly for patients with (myxoid) lipo- and leiomyosarcomas [3,4].

More recently, the phase III PALETTE study [5] introduced pazopanib (Votrient®) as treatment for adult patients with non-lipomatous advanced STS who have received prior chemotherapy for metastatic disease or who have progressed within 12 months after (neo)adjuvant therapy. This was based on a progression free survival of 4.6 months for pazopanib versus 1.6 months in the placebo arm. Pazopanib is an oral anti-angiogenic multi-targeted tyrosine kinase inhibitor with activity against vascular endothelial growth factor receptors (VEGFR) 1, 2 and 3, platelet derived growth factor receptors and KIT. In the PALETTE study, treatment with pazopanib was complicated by the occurrence of a pneumothorax in 8 of the 246 patients. This case series reports on the incidence and clinic of this complication in all consecutive patients treated at the Leiden University Medical Centre.

## Cases

**Figure 1:**
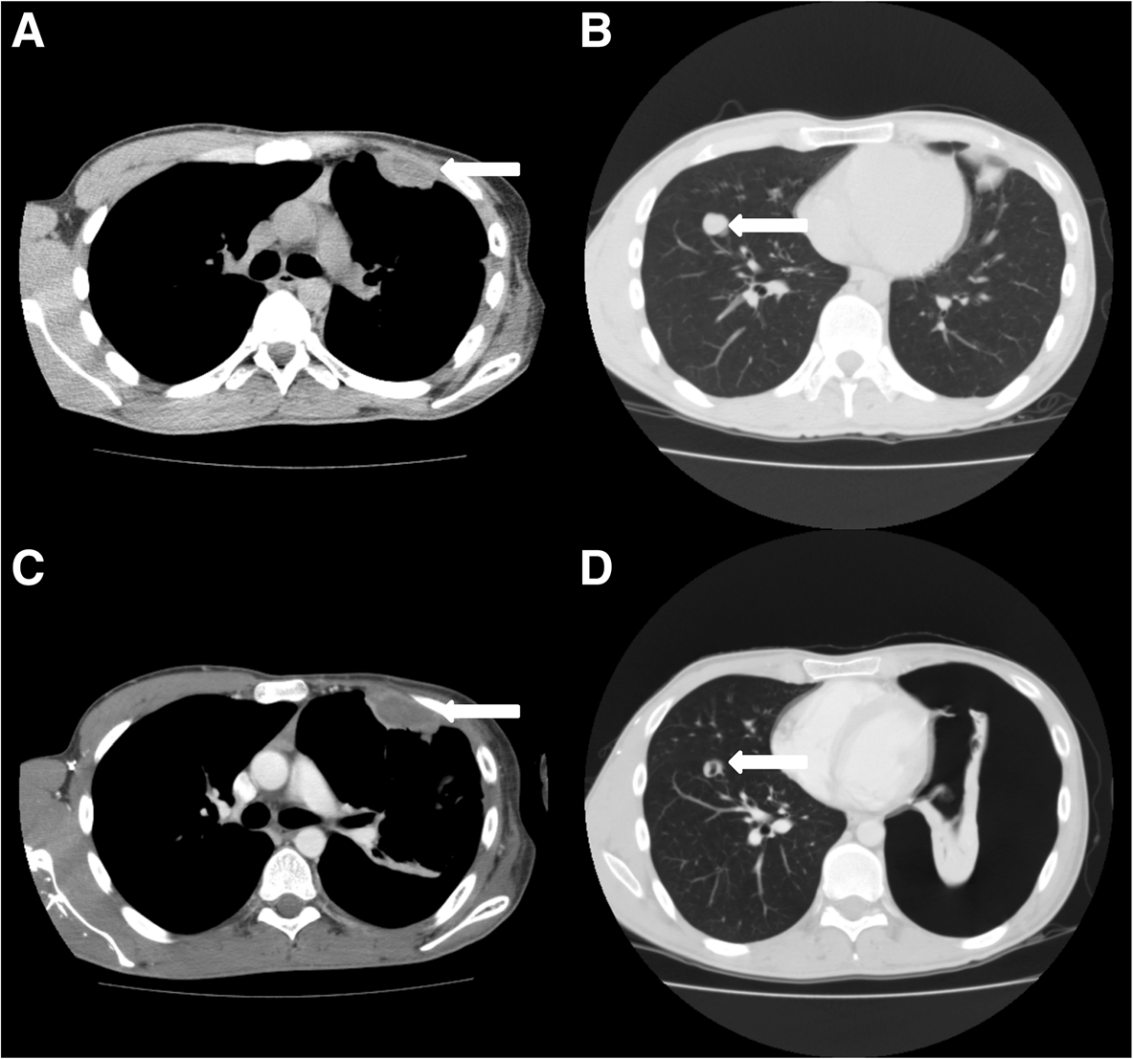
**CT scans of patient 1. A** and **B**: CT scan of the thorax at start of pazopanib, showing a pleural metastasis of the left lung with central hypodensities suggesting necrosis (A, arrow) and showing a pulmonary metastasis in the right lung (B, arrow). **C** and **D**: CT scan, when presenting with a left-sided pneumothorax **(D)**, showing progressive hypodensity of the pleural metastasis on the left side (C, arrow) and central cavitation in the metastasis in the right lung (D, arrow).

**Figure 2:**
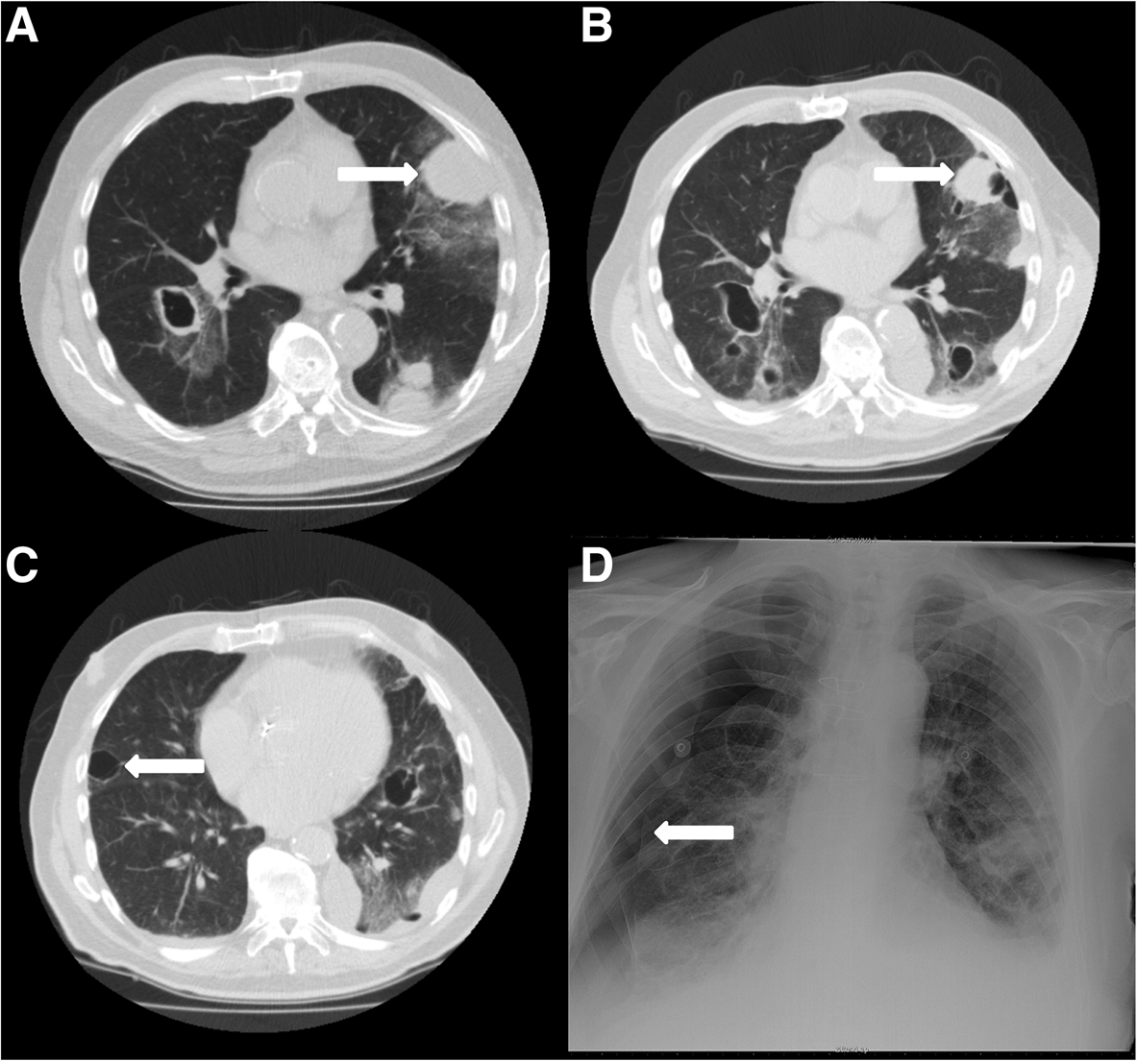
**CT scans and X-thorax of patient 2. A**: CT scan at start of pazopanib showing metastases in both lungs with a large necrotizing metastasis on the left side. The pleural metastasis indicated by the arrow is also visible in **B**, which is a CT scan after 3 months of pazopanib treatment, now showing cavitations. **C** is also an image from the CT scan after 3 months of pazopanib treatment showing a metastasis (arrow) with cavitation next to the pleura. **D**: The X-thorax shows the right sided pneumothorax. The visceral pleural line is indicated by the arrow.

**Figure 3:**
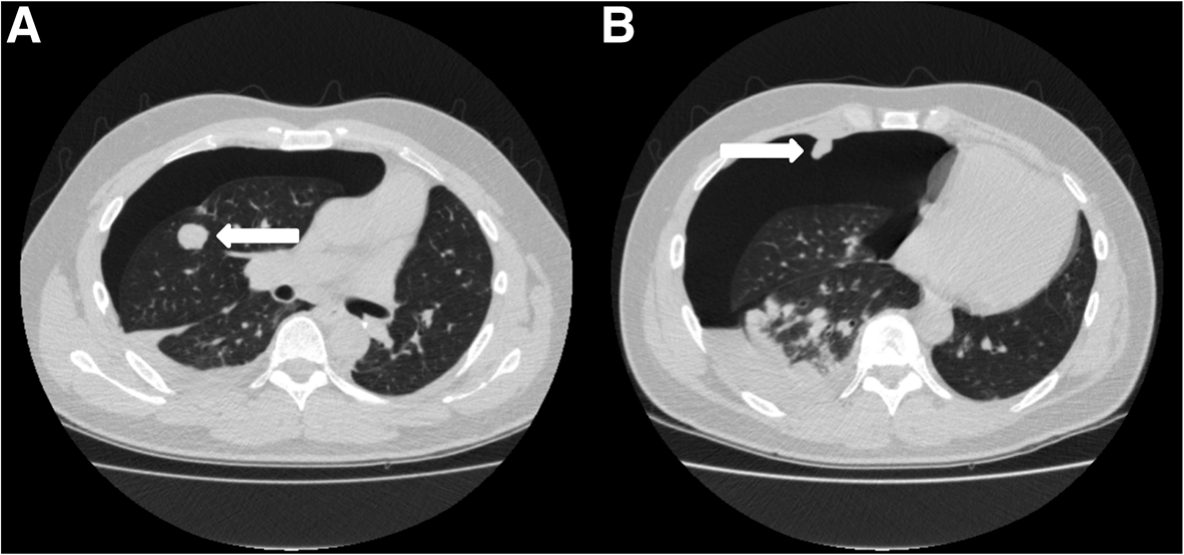
**CT scans of patient 3.** Routine follow-up CT scan showing the pneumothorax on the right side. **A** shows one of the pulmonary metastasis, **B** shows a pleural metastasis.

**Figure 4:**
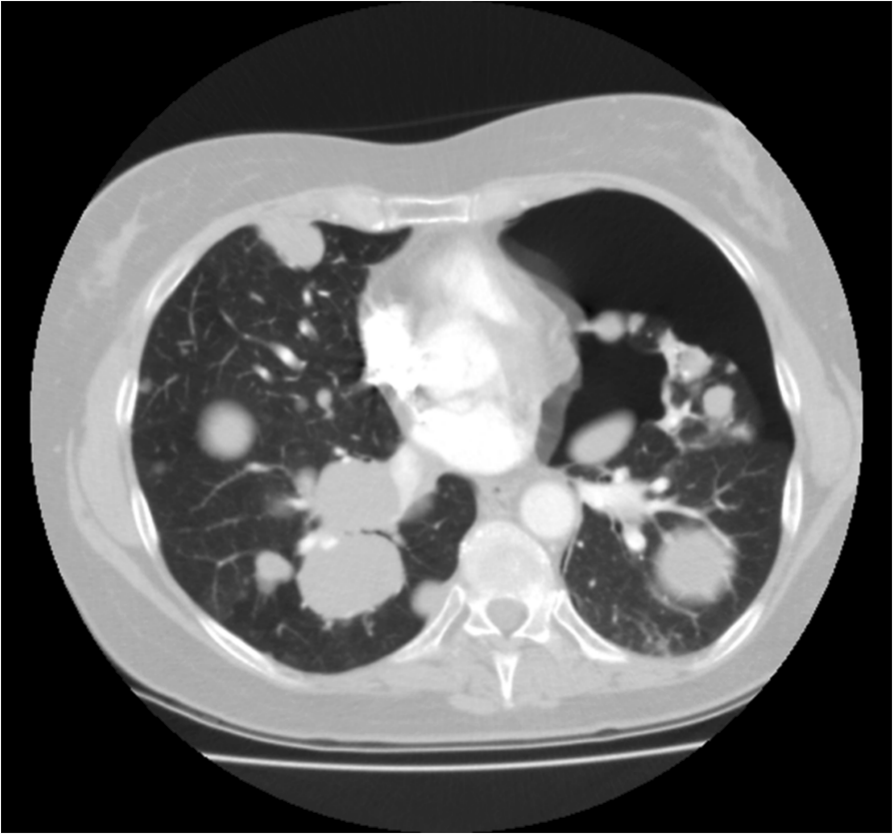
**CT scan of patient 4.** The CT scan shows the massive pulmonary metastases and a left-sided pneumothorax.

**Figure 5:**
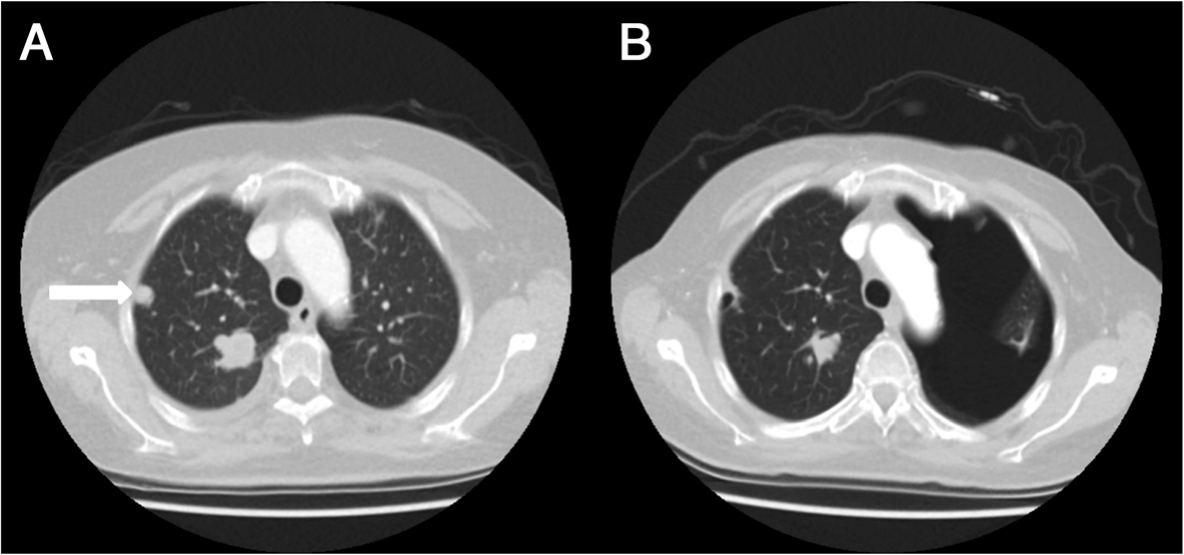
**CT scans of patient 5. A**: CT scan before the start of pazopanib showing multiple metastases in the right lung, one is indicated by the arrow. **B**: Routine follow-up CT scan during pazopanib treatment showing the left-sided pneumothorax and the earlier mentioned metastasis in the right lung is now showing cavitation.

**Figure 6:**
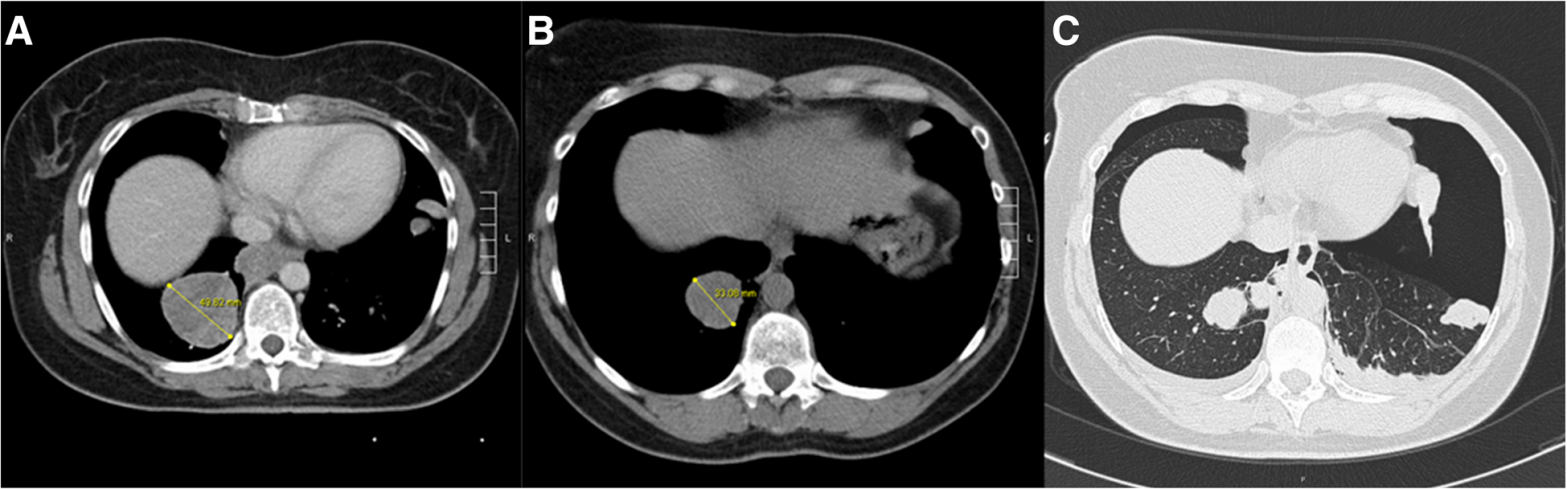
**CT scan of patient 6. A**: CT scan at start of pazopanib showing a metastasis in the right lower lobe with a diameter of 49.6 mm. **B**: CT scan after 12 weeks of pazopanib (6 weeks of treatment and 6 weeks on hold due to liver toxicity), showing a decrease in diameter of the metastasis to 33.1 mm. **C**: CT scan showing the bilateral pneumothorax.

In our institution, 43 STS patients were treated with pazopanib, of which 39 had pulmonary metastases and 36 of these patients had pleural or subpleural pulmonary metastases. Treatment was complicated by a pneumothorax in six (14.0%) patients.The first patient is a 24-year old male, with a malignant peripheral nerve sheath tumour of the left brachial plexus, diagnosed three years before. Initial treatment consisted of local resection and irradiation, repeated one year later because of local recurrence. Pulmonary metastases, some localised pleural, were found seven months later for which six cycles of 3-weekly doxorubicin 75 mg/m2 was initiated. Progressive disease was diagnosed six months after the first doxorubicin cycle and treatment with pazopanib was started. A CT scan at the start of pazopanib showed a necrotizing metastasis in the left lung (Figure 1A, B). A month later a left-sided pneumothorax occurred after a skiing trip at 3000 meters height, persisting after drainage and later also a right sided pneumothorax occurred. The CT scan detecting the pneumothorax on the left side, also showed cystic degeneration of metastases in the right lower lobe (Figure 1C, D). The persistent bilateral pneumothorax was complicated by a pyothorax on the left side. He died six months after the start of pazopanib, which was continued until his death.Patient 2 is a 79-year old male, who was diagnosed two years before with an angiosarcoma of the scalp. Primary treatment was combined paclitaxel and radiotherapy. However, one year later a local recurrence was diagnosed and a CT scan showed pulmonary metastases, of which some were pleural, for which 3-weekly doxorubicin 75 mg/m2 was started. The local recurrence was progressive after six months and pazopanib was initiated, which was stopped three months thereafter because of progressive disease. Some of the pulmonary metastases already showing cavitation at start of pazopanib (Figure 2A) were increasing in number, size and cavitation during treatment (Figure 2B, C). One week after the start of pazopanib a right sided pneumothorax occurred (Figure 2D), which was treated with drainage, but without pleurodesis, however it recurred one week later and again drained. A month thereafter a left-sided pneumothorax was diagnosed, which was left untreated. He died one week later due to disease progression, four months after the start of pazopanib.The third patient is a 34-year old male, three years before diagnosed with a synovial sarcoma of the left femur with synchronous lung metastases, some with a pleural localisation, and malignant pleural effusion. Treatment consisted of resection of the primary tumour and doxorubicin/ifosfamide chemotherapy followed by pulmonary metastasectomy and isolated melphalan lung perfusion. Treatment with trabectedin was started, but stopped after nine cycles because of progressive disease and pazopanib was started. He was treated for a remarkable 15 months when progression occurred and a hydropneumothorax was diagnosed on a routine follow-up CT scan (Figure 3A, B), treated with drainage and talc pleurodesis. Pazopanib was stopped. The CT scan did not show necrotizing metastases. Two weeks later the pleural effusion recurred, and persisted until he died 2 months later.The fourth patient is a 53-year old female, who had resection of a low-grade fibromyxomatoid sarcoma of the left lower extremity 13 years before. Nine years later, pulmonary, liver and lymph node metastases were diagnosed, treated with 2 cycles of liposomal doxorubicin, which was stopped because of toxicity. Some of the pulmonary metastases were localised adjacent to the pleura. Treatment was continued with low dose doxorubicin weekly for 3 months, which was repeated one year later because of progressive disease. Three months after the last doxorubicin treatment progressive disease was diagnosed and pazopanib was prescribed. After seven months treatment with pazopanib she developed a left-sided pneumothorax (Figure 4), successfully treated with drainage. No cavitation of the metastases was found. At the moment, treated for 31 months with pazopanib, she has stable disease.Patient five is a 72-year old female, who presented with a high grade undifferentiated pleomorphic sarcoma of the right lower extremity with pulmonary metastases, of which some are localised next to the pleura, one year before. She was treated with local resection, radiotherapy and 3-weekly doxorubicin 75 mg/m2. Five months after the start of doxorubicin progressive disease was diagnosed and pazopanib was prescribed (Figure 5A). After two months of treatment she developed a left-sided pneumothorax, treated with drainage and pleural rubbing (Figure 5B). Part of the pulmonary metastases showed cavitation. During admission a right-sided pneumothorax occurred and was drained successfully. However, she died one week later due to progressive disease.The sixth patient is a 40-year old female diagnosed with a low grade endometrial stromal sarcoma, six years before, after a uterus extirpation for uterine myomatosis. When she presented four years later with thoracic pain, CT scan of the thorax showed a large mediastinal mass and pulmonary metastases, also localised next to the pleural space. Treatment with doxorubicin was started and resulted in partial regression of the tumour masses. Eighteen months later pazopanib was started under the supervision of the reference centre because of progression of the pulmonary metastases. Due to liver toxicity the pazopanib dose was tapered to a minimum dose of 200 mg with short pauses in treatment, however treatment was successful (Figure 6A, B). Treatment was permanently stopped after 33 months because of a bilateral pneumothorax and disease progression (Figure 6C). She was successfully treated with drainage.

## Discussion

The incidence of pneumothorax of 14.0% in our institution was higher than the previous reported incidence (3.3%) in the PALETTE trial [5]. Literature does not provide the incidence of secondary spontaneous pneumothorax in STS patients, but it is probably uncommon. Other trials with pazopanib in for example urothelial cancer, renal cell cancer, pancreatic neuroendocrine tumours and cervical cancer did not report pneumothorax as an adverse event [6-11]. Trials with sunitinib, another VEGFR inhibitor, in renal cell carcinoma also do not report pneumothorax as an adverse event [12,13]. Only, two cases are published in literature of patients with a spontaneous pneumothorax during sunitinib treatment for renal cell carcinoma [14,15]. This suggests that this is a specific adverse event in metastatic STS treated with pazopanib, however, it could still be due to underreporting in other studies or due to the natural course of this disease that predominantly metastasizes to the lungs.

One of the proposed mechanisms is necrosis of a metastasis due to therapy, resulting in a pleural defect. Another possible explanation would be a check valve mechanism due to compression of bronchioles by a lung metastasis causing hyperinflation of a lung segment and rupture of lung parenchyma [16]. In our patients all cases of pneumothorax were related to pleural or subpleural lung metastases, and were observed in both progressive and responding patients. Smoking is a risk factor for primary spontaneous pneumothorax, but only one of our six patients had a smoking history. We do not think smoking history is relevant for the occurrence of a pneumothorax in these patients. As with other cancer related pneumothoraxes they were difficult to treat. One of the explanations for the difficult treatment in these patients could be the use of pazopanib, which inhibits angiogenesis and thereby tissue regeneration.

A larger series is needed in a case control setting to gain more understanding of this phenomenon.

## Conclusion

The risk of a difficult to treat pneumothorax during pazopanib therapy should be discussed with the patient before initiation of treatment for a pulmonary metastasized sarcoma and physicians should be alert to the occurrence of such an event.

## Consent

Written informed consent was obtained from every patient in the PALETTE study or the pazopanib compassionate use program for use of their medical data for scientific purposes. A copy of the written informed consent is available for review by the Editor-in-Chief of this journal.

## Abbreviations

STS: Soft tissue sarcoma; VEGFR: Vascular endothelial growth factor receptor.

## Competing interests

The authors declare that they have no competing interests.

## Authors’ contributions

AJV participated in the collection of the data and the literature search and drafted the manuscript. HG participated in the design of the study and corrected the manuscript. Both authors have read and approved the final manuscript.

## References

[B1] ESMO/European Sarcoma Network Working Group Soft tissue and visceral sarcomas: ESMO clinical practice guidelines for diagnosis, treatment and follow-up Ann Oncol 2012 23 Suppl 7 vii92 vii99 22997462 10.1093/annonc/mds253

[B2] Sleijfer S Seynaeve C Verweij J Using single-agent therapy in adult patients with advanced soft tissue sarcoma can still be considered standard care Oncologist 2005 10 10 833 841 10.1634/theoncologist.10-10-833 16314294

[B3] Le Cesne A Blay JY Judson I Van Oosterom A Verweij J Radford J Lorigan P Rodenhuis S Ray-Coquard I Bonvalot S Collin F Jimeno J Di Paola E Van Glabbeke M Nielsen OS Phase II study of ET-743 in advanced soft tissue sarcomas: a European Organisation for the Research and Treatment of Cancer (EORTC) soft tissue and bone sarcoma group trial J Clin Oncol 2005 23 3 576 584 15659504 10.1200/JCO.2005.01.180

[B4] Demetri GD Chawla SP von Mehren M Ritch P Baker LH Blay JY Hande KR Keohan ML Samuels BL Schuetze S Lebedinsky C Elsayed YA Izquierdo MA Gomez J Park YC Le Cesne A Efficacy and safety of trabectedin in patients with advanced or metastatic liposarcoma or leiomyosarcoma after failure of prior anthracyclines and ifosfamide: results of a randomized phase II study of two different schedules J Clin Oncol 2009 27 25 4188 4196 10.1200/JCO.2008.21.0088 19652065

[B5] van der Graaf WT Blay JY Chawla SP Kim DW Bui-Nguyen B Casali PG Schoffski P Aglietta M Staddon AP Beppu Y Le Cesne A Gelderblom H Judson IR Araki N Ouali M Marreaud S Hodge R Dewji MR Coens C Demetri GD Fletcher CD Dei Tos AP Hohenberger P EORTC Soft Tissue and Bone Sarcoma Group; PALETTE study group Pazopanib for metastatic soft-tissue sarcoma (PALETTE): a randomised, double-blind, placebo-controlled phase 3 trial Lancet 2012 379 9829 1879 1886 10.1016/S0140-6736(12)60651-5 22595799

[B6] Friedlander M Hancock KC Rischin D Messing MJ Stringer CA Matthys GM Ma B Hodge JP Lager JJ A Phase II, open-label study evaluating pazopanib in patients with recurrent ovarian cancer Gynecol Oncol 2010 119 1 32 37 10.1016/j.ygyno.2010.05.033 20584542

[B7] Necchi A Mariani L Zaffaroni N Schwartz LH Giannatempo P Crippa F Morosi C Lanocita R Sava T Ortega C Messina C Sacco C Pennati M Daidone MG Nicolai N De Braud F Gianni AM Salvioni R Pazopanib in advanced and platinum-resistant urothelial cancer: an open-label, single group, phase 2 trial Lancet Oncol 2012 13 8 810 816 10.1016/S1470-2045(12)70294-2 22819172

[B8] Sternberg CN Hawkins RE Wagstaff J Salman P Mardiak J Barrios CH Zarba JJ Gladkov OA Lee E Szczylik C McCann L Rubin SD Chen M Davis ID A randomised, double-blind phase III study of pazopanib in patients with advanced and/or metastatic renal cell carcinoma: final overall survival results and safety update Eur J Cancer 2013 49 6 1287 1296 10.1016/j.ejca.2012.12.010 23321547

[B9] Rautiola J Utriainen T Peltola K Joensuu H Bono P Pazopanib after sunitinib failure in patients with metastatic renal cell carcinoma Acta Oncol 2014 53 1 113 118 10.3109/0284186X.2013.794957 23721300

[B10] Pili R Qin R Flynn PJ Picus J Millward M Ho WM Pitot H Tan W Miles KM Erlichman C Vaishampayan U A phase II safety and efficacy study of the vascular endothelial growth factor receptor tyrosine kinase inhibitor pazopanib in patients with metastatic urothelial cancer Clin Genitourin Cancer 2013 11 4 477 483 10.1016/j.clgc.2013.05.005 23891158 PMC4102930

[B11] Ahn HK Choi JY Kim KM Kim H Choi SH Park SH Park JO Lim HY Kang WK Lee J Park YS Phase II study of pazopanib monotherapy in metastatic gastroenteropancreatic neuroendocrine tumours Br J Cancer 2013 109 6 1414 1419 10.1038/bjc.2013.470 23989950 PMC3776983

[B12] Motzer RJ Hutson TE Tomczak P Michaelson MD Bukowski RM Rixe O Oudard S Negrier S Szczylik C Kim ST Chen I Bycott PW Baum CM Figlin RA Sunitinib versus interferon Alfa in metastatic renal-cell carcinoma N Engl J Med 2007 356 2 115 124 10.1056/NEJMoa065044 17215529

[B13] Gore ME Szczylik C Porta C Bracarda S Bjarnason GA Oudard S Hariharan S Lee S-H Haanen J Castellano D Vrdoljak E Schöffski P Mainwaring P Nieto A Yuan J Bukowski R Safety and efficacy of sunitinib for metastatic renal-cell carcinoma: an expanded-access trial Lancet Oncol 2009 10 8 757 763 10.1016/S1470-2045(09)70162-7 19615940

[B14] Katta A Fesler MJ Tan A Vuong G Richart JM Spontaneous bilateral pneumothorax in metastatic renal cell carcinoma on sunitinib therapy Cancer Chemother Pharmacol 2010 66 2 409 412 10.1007/s00280-010-1291-3 20204363

[B15] Kleontas A Asteriou C Lalountas M Konstantinou E Barbetakis N Spontaneous pneumothorax complicating sunitinib therapy Hippokratia 2011 15 3 281 282 22435034 PMC3306043

[B16] Lee M-J Kim E-K Kim MJ Kwak JY Hong S Park CS Spontaneous pneumothorax in metastatic thyroid papillary carcinoma J Clin Oncol 2007 25 18 2616 2618 10.1200/JCO.2007.11.0130 17577042

